# Insights From Deep Sequencing of the HBV Genome—Unique, Tiny, and Misunderstood

**DOI:** 10.1053/j.gastro.2018.07.058

**Published:** 2019-01

**Authors:** Anna L. McNaughton, Valentina D’Arienzo, M. Azim Ansari, Sheila F. Lumley, Margaret Littlejohn, Peter Revill, Jane A. McKeating, Philippa C. Matthews

**Affiliations:** 1 Nuffield Department of Medicine, Peter Medawar Building for Pathogen Research, Oxford, United Kingdom; 2 Nuffield Department of Medicine, NDM Research Building, Oxford, United Kingdom; 3 Department of Infectious Diseases and Microbiology, Oxford University Hospitals NHS Foundation Trust, John Radcliffe Hospital, Oxford, United Kingdom; 4 Victorian Infectious Diseases Reference Laboratory, Royal Melbourne Hospital at the Peter Doherty Institute of Infection and Immunity, Melbourne, Australia; 5 Department of Microbiology and Immunology, University of Melbourne. Melbourne, Australia

**Keywords:** Hepatitis B Virus, Genotype, Diversity, Evolution, cccDNA, covalently closed circular DNA, dsDNA, double-stranded DNA, HBsAg, hepatitis B surface antigen, HBeAg, hepatitis B e antigen, HBV, hepatitis B virus, HCC, hepatocellular carcinoma, HIV, human immunodeficiency virus, NGS, next-generation sequencing, ORF, open reading frame, P, reverse transcriptase polymerase, RC-DNA, relaxed circular DNA, RT, reverse transcriptase, S, surface

## Abstract

Hepatitis B virus (HBV) is a unique, tiny, partially double-stranded, reverse-transcribing DNA virus with proteins encoded by multiple overlapping reading frames. The substitution rate is surprisingly high for a DNA virus, but lower than that of other reverse transcribing organisms. More than 260 million people worldwide have chronic HBV infection, which causes 0.8 million deaths a year. Because of the high burden of disease, international health agencies have set the goal of eliminating HBV infection by 2030. Nonetheless, the intriguing HBV genome has not been well characterized. We summarize data on the HBV genome structure and replication cycle, explain and quantify diversity within and among infected individuals, and discuss advances that can be offered by application of next-generation sequencing technology. In-depth HBV genome analyses could increase our understanding of disease pathogenesis and allow us to better predict patient outcomes, optimize treatment, and develop new therapeutics.

**Figure:**
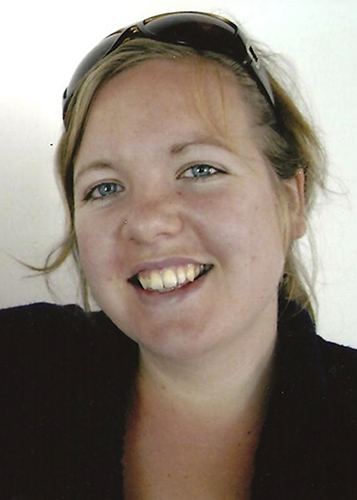
Anna L. McNaughton

**Figure:**
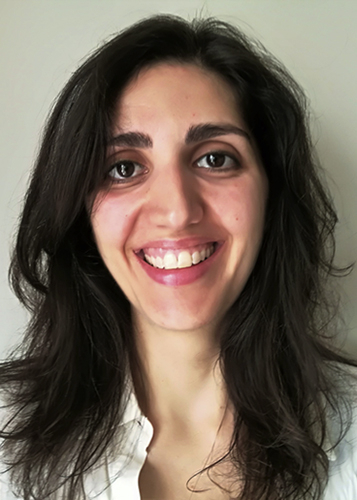
Valentina D’Arienzo

**Figure:**
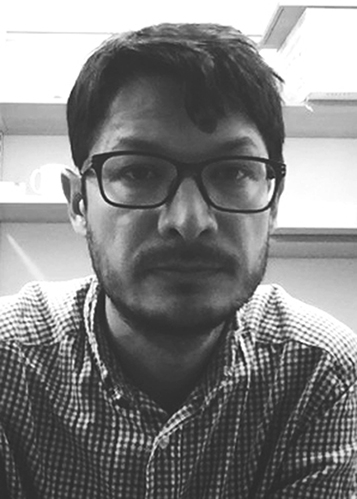
M. Azim Ansari

**Figure:**
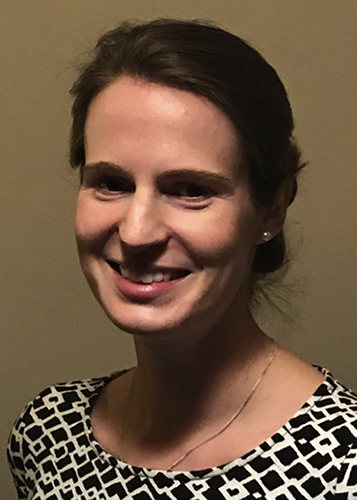
Sheila F. Lumley

**Figure:**
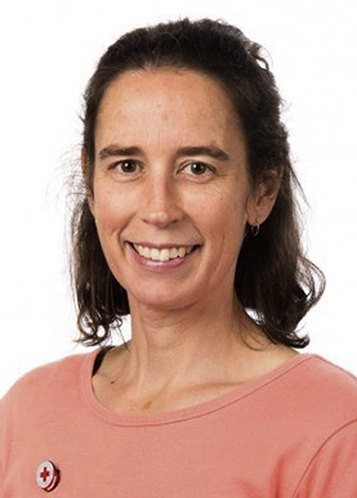
Margaret Littlejohn

**Figure:**
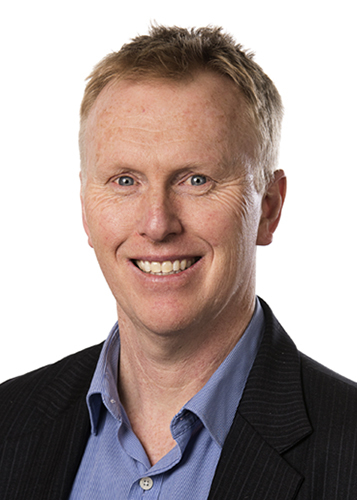
Peter Revill

**Figure:**
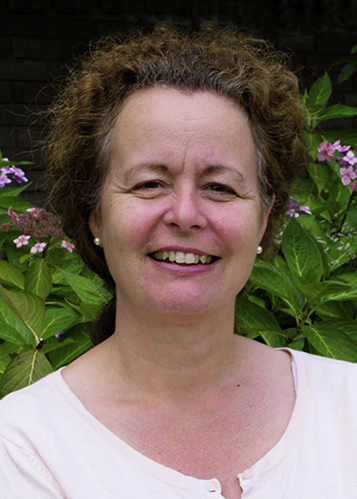
Jane A. McKeating

**Figure:**
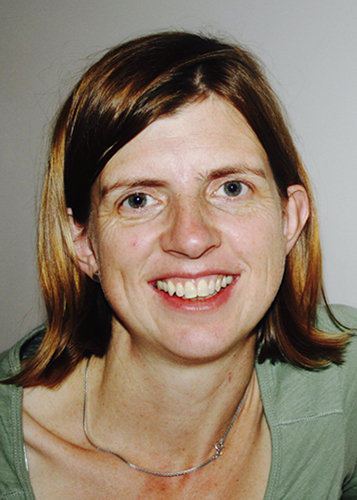
Philippa C. Matthews

Hepatitis B virus (HBV) was first identified in the 1960s by Baruch Blumberg, who went on to win the Nobel prize for this discovery.^1^, ^2^ The virus is a leading cause of liver disease worldwide: an estimated 250–260 million individuals are chronically infected, and approximately one third of the world’s population has serologic evidence of exposure.^3^ HBV is a global public health problem with endemic levels of infection in Southeast Asia and Africa, where prevalence rates are at least 8% in many populations.^4^, ^5^ However, HBV is under-represented in terms of resource allocation, political advocacy, and research.^6^

Chronic HBV infection leads to liver inflammation, with long-term risks of cirrhosis and hepatocellular carcinoma (HCC).^7^, ^8^ In contrast to the decrease in mortality from human immunodeficiency virus (HIV), tuberculosis, and malaria, HBV-associated mortality is increasing.^9^ The United Nations Sustainable Development Goals set the challenge of eliminating HBV infection as a public health threat by 2030.^10^, ^11^ However, substantial barriers to elimination include gaps in vaccine coverage, long periods between vaccination and its effects on population prevalence,^12^ and lack of a cure. Other challenges include the virus’s resistance to drugs (and to a lesser extent vaccines),^13^, ^14^ HIV coinfection, stigma, poverty, lack of education, and limited access to diagnostic tests.^6^ HBV infection is treated with interferon and nucleos(t)ide analogue reverse transcriptase (RT) inhibitors—primarily tenofovir or entecavir—which can limit liver damage by suppressing viral replication.^15^ However, interferon therapy is associated with unpleasant side effects and cures only a small percentage of patients. Nucleos(t)ide analogue RT inhibitors decrease viremia but have no consistent effect on clearance. Therefore, rebound viremia after cessation is common. There is a great need to cure HBV infection if we are to achieve elimination targets; curative therapy for HBV is an important goal for individual patients and the international public health agenda.^16^

Curing HBV infection requires a detailed and robust understanding of the genetic sequence, structure, and diversity of HBV. Scientific investment is required to develop panels of diverse infectious clones that replicate in cell lines and in animals, to support drug resistance-screening programs.^17^ Detailed insights into immune control and clearance can be gained from identifying sites of immune selection pressure in the virus genome.^18^ This approach has helped identify immune correlates of HIV control over the past decade.^18^, ^19^ Increasing our understanding of virus genetics can improve management of patients—in stratification, selection of therapy, identification of drug- and vaccine-resistant strains, and development of new approaches to monitoring.^20^

HBV sequence data largely consist of consensus sequences of individual viral genes derived by Sanger sequencing. However, next-generation sequencing (NGS) platforms are rapidly becoming more accessible and affordable, in addition to new bioinformatic approaches to handle the resulting datasets.^21^, ^22^, ^23^ In addition to enabling whole-genome sequencing, NGS offers a powerful method for detection of minor variants relevant to the identification of drug resistance,^24^, ^25^, ^26^ studies of quasispecies dynamics,^27^ and characterization of complex viral populations.^28^ Together with improved curation and publication of clinical metadata, these accurate, full-length, ultra-deep HBV sequence data provide increasing opportunities for developing new insights into HBV evolution, diversity, pathogenesis, immune control, and treatment outcomes.

To provide a solid foundation for interpretation of new sequence datasets, we assimilate available data on HBV genome structure, function, and diversity and summarize gains made using NGS platforms.

## Taxonomy

**Figure 1:**
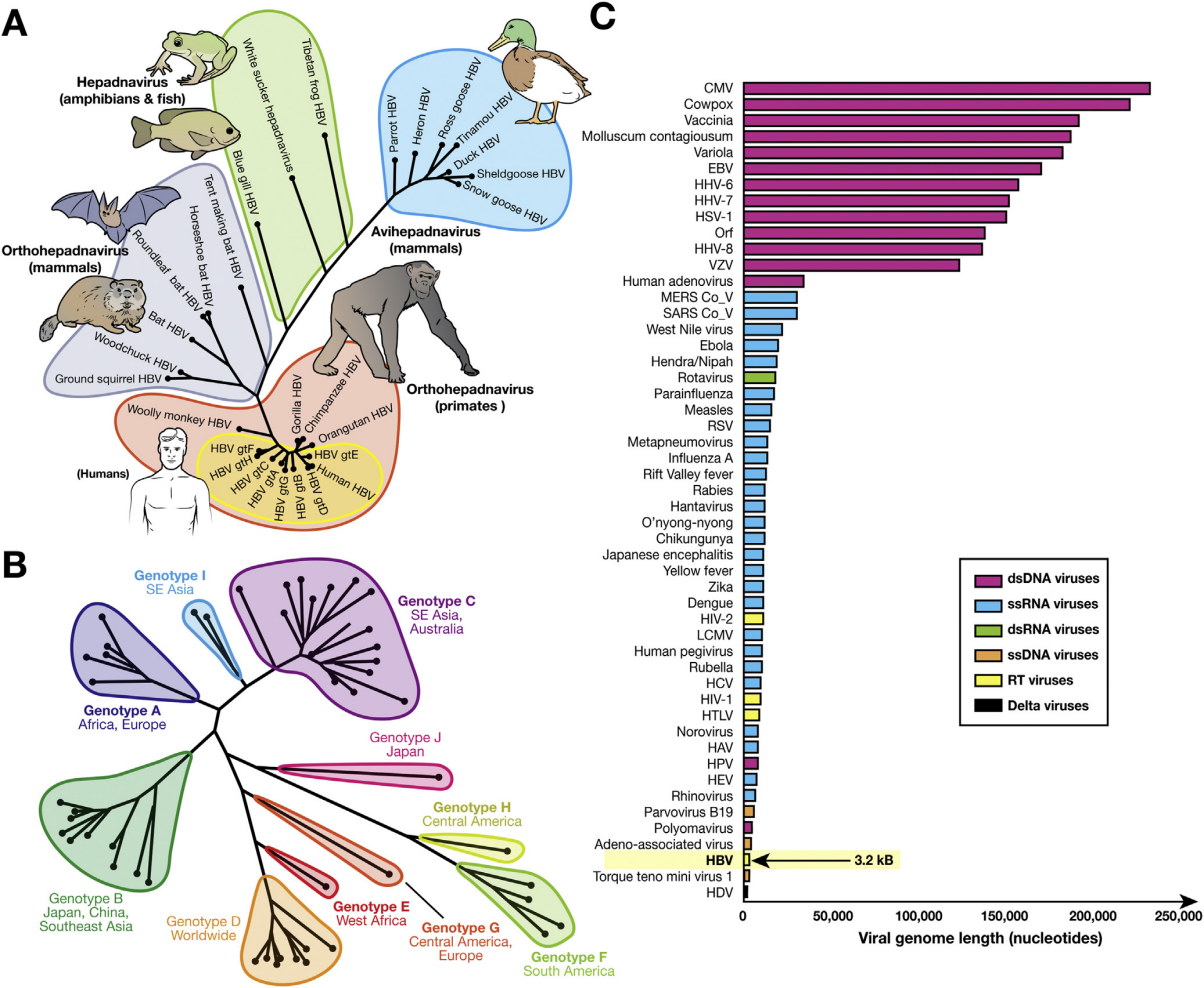
Relationships between HBV and other hepadnaviruses, genotype diversity, and genome size. (*A*) Phylogenetic tree of the relation among avian, mammalian, and other hepadnaviruses. Hepadnavirus reference sequences for avian (NC_005950.1, NC_001344.1, NC_016561.1, NC_005890.1, NC_001486.1, NC_035210.1, NC_005888.1), mammalian (NC_003977.2, NC_028129.1, NC_024445.1, NC_024444.1, NC_024443.1, NC_020881.1, NC_004107.1, NC_001484.1), and other (NC_027922.1, NC_030446.1, NC_030445.1) species were downloaded from Genbank.^32^ This dataset was further supplemented with hepadnavirus isolates from chimpanzees, orangutans, and gorillas (AF193863, FJ798097, FJ798098) and some widely cited HBV genotype strains (X02763, D00330, AY123041, V01460, X75657, X69798, AF160501, AY090454). (*B*) Midpoint-rooted maximum likelihood phylogenetic tree generated using MEGA7^33^ with bootstrap replicates of 1000 used, indicating relations between HBV genotypes and subtypes and their typical geographic distribution. Widely used reference sequences for genotypes A–D and F are included. For genotypes with a single subtype, the reference sequences were used to generate the tree. The sequences used to generate the tree were genotype A: KP234050.1, HE974376.1, KP234052.1, AY934764.1, KP234053.1, GQ331048.1; genotype B: D50521.1, AB073825.1, GQ924628.1, AB073826.1, AB219427.1, DQ463792.1, AP011091.1, AP011093.1, GQ205440.1, GQ358146.1; genotype C: KM999990.1, KY629637.1, AB554019.1, AB554018.1, AB644281.1, AB644283.1, AB644286.1, AB644287.1, KP017272.1, KU695741.1, KF873519.1, KM999992.1, KM999993.1, AP011107.1, KP017269.1, AP011108.1; genotype D: AB104711.1, HQ700511.1, KP090181.1, FJ692533.2, DQ315780.1, KF170740.1, KP322600.1, FJ904406.1; genotype F: AF223963.1, AY311369.1, AY311370.1, AB166850.1; genotype I: AB562462.1, FJ023671.1; genotype J: AB486012.1; and HBVdb genotype reference sequences for genotypes A–H, respectively: X02763, D00331, AY123041.1, V01460.1, X75657.1, X69798.1, AF160501, AY090454. (*C*) Relative genome sizes of viruses pathogenic to humans including HBV (3.2 kB; *arrow*). Genomes were obtained from https://www.ncbi.nlm.nih.gov/genomes/GenomesGroup.cgi?taxid=10239 and sorted by nucleotide length. For each virus type, a representative genome was selected. Metadata, including accession numbers for each organism, can be found at 10.6084/m9.figshare.6080402. CMV, cytomegalovirus; dsRNA, double-stranded RNA; EBV, Epstein-Barr virus; gt, genotype; HAV, hepatitis A virus; HCV, hepatitis C virus; HDV, hepatitis D virus; HEV, hepatitis E virus; HHV, human herpesvirus; HPV, human papillomavirus; HSV, herpes simplex virus; HTLV, human T-lymphotropic virus; LCMV, lymphocytic choriomeningitis virus; MERS, Middle East Respiratory Syndrome; SARS, Severe Acute Respiratory Syndrome; ssDNA, single-stranded DNA; ssRNA, single-stranded RNA; VZV, varicella zoster virus.

HBV is the prototype virus of the Hepadnaviridae family—small spherical viruses with icosahedral symmetry that combine a partial double-stranded (ds) DNA genome and virus-encoded RT. Within the Baltimore virus classification system, which classifies viruses based on their genomic composition and replication cycles,^29^ the Hepadnaviridae are classified as group VII (sometimes referred to as pararetroviruses)—they are the only animal viruses of this group. Until recently, the family was divided into 2 genera: the *Orthohepadnavirus* species (which infect mammals, including primates and bats) and the *Avihepadnavirus* species (which infect birds). However, the recent discovery of putative hepadnaviruses that infect fish^30^ and amphibians^31^ indicates that the viral family might be larger than initially believed (Figure 1*A*).^32^, ^33^ Based on sequence diversity, HBV is divided into 9 genotypes and 1 putative genotype (Figure 1*B*). Hepadnaviruses have some of the smallest known viral genomes, ranging from 3.0 to 3.3 kb; the HBV genome is approximately 3.2 kb^34^ (Figure 1*C*).

## Genome Structure

**Figure 2:**
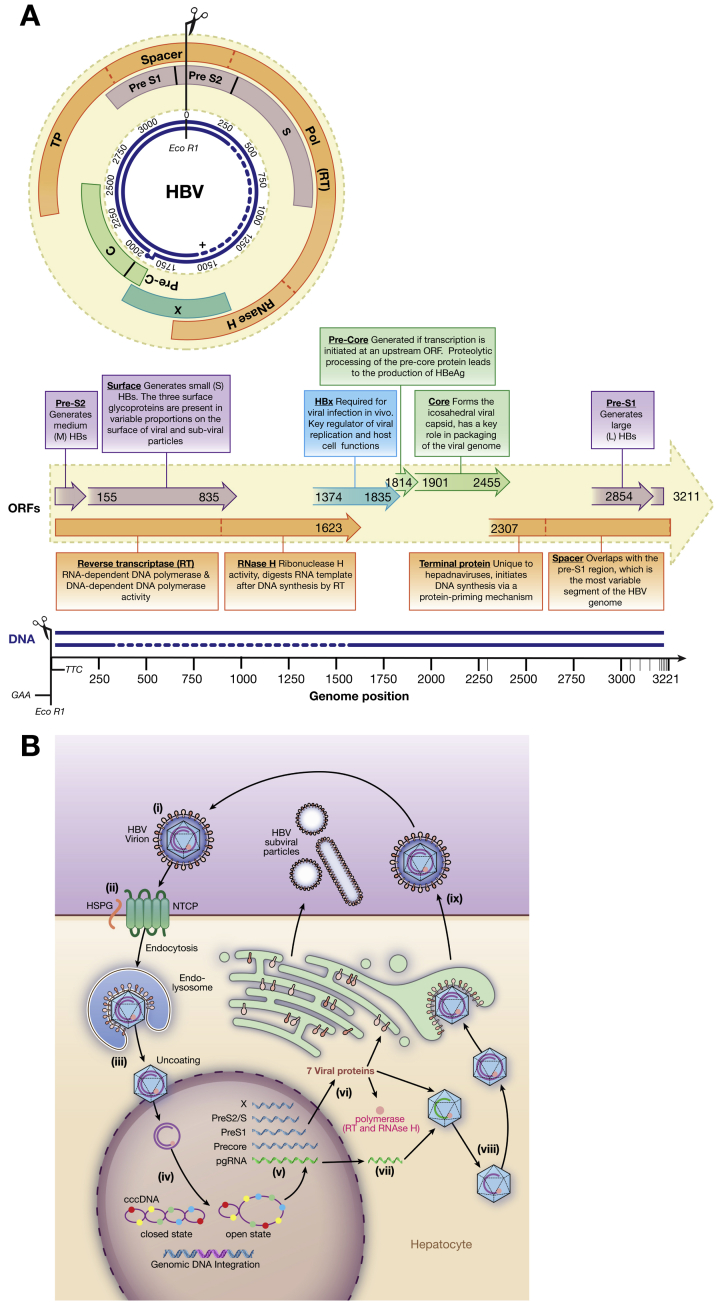
Annotated HBV genome and replication cycle. (*A*) The 4 overlapping ORFs and the 7 products encoded. Gene products are indicated by text boxes, with start and end positions derived using X02763.1 as a reference strain. The major functional domains of the P gene product are indicated (*dotted lines*). Large HBs consist of pre-S1, pre-S2, and S; medium HBs consist of pre-S2 and S; and small HBs consist of S only. The overlap of >1000 nucleotides between the P and S genes is the largest gene overlap of any known animal virus.^35^ The near-complete negative DNA strand and partially complete positive DNA strands (*dotted line* indicates approximated missing region) also are shown, in addition to the position of *EcoR1*. The 5′ end of the complete negative-sense DNA strand is covalently bound to the viral RT. The complementary positive-sense DNA strand is partially complete, covering approximately two thirds of the viral genome.^36^ The 5′ end of the incomplete strand is defined by a short oligo-ribonucleotide region; the 3′ end varies within and among hosts. (*B*) Replication cycle (adapted from Liang, special issue). (*i*) Infective HBV virions in serum, often referred to as Dane particles (diameter, 42 nm). The capsid structure has icosahedral symmetry: T = 4 (31 nm; 90% of population) and T = 3 (28 nm; 10% of population).^37^, ^38^ (*ii*) The virus enters hepatocytes by HSPG (low-affinity binding) and solute carrier family 10 member 1 (SLC10A1; also called sodium taurocholate co-transporting polypeptide NTCP; high-affinity binding). (*iii*) The molecular processes of un-coating and nuclear import are unclear but likely require cell proteins. (*iv*) Viral DNA enters the nucleus as RC-DNA. (*v*) Viral DNA is reconfigured as cccDNA within the nucleus by the cell’s DNA repair factors; this stable structure occurs in association with host histones that mediate DNA packaging. (*vi*) The open cccDNA structure is a template for host RNA polymerase II. (*vii*) DNA is transcribed to pre-genomic RNA intermediates in the nucleus, creating 4 mRNAs (*blue*): a 3.5-kb transcript encoding precore RNA (full-length pre-genomic RNA also shown in *green*); 2.4- and 2.1-kb mRNA transcripts for pre-S and S, respectively; and a 0.7-kb mRNA encoding the X protein. The RNA is transported to the cytoplasm, where it is translated to 7 viral proteins (short, medium, and long S proteins, core, e antigen, polymerase, and X protein). (*viii*) HBV RT produces a negative-strand DNA from pre-genomic RNA. The RNA template is degraded by RNase H, and then synthesis of the positive-strand DNA is initiated. HBV DNA is repackaged in relaxed form with other proteins inside the host cell. (*ix*) New virions and viral proteins are released into the blood. Excess HBsAg forms small noninfectious, subviral particles (∼20 nm diameter), and long filaments^161^; free HBeAg and capsids also are secreted. C, core; HBeAg, hepatitis B e antigen; HBx, hepatitis B X protein; HSPG, heparan sulfate proteoglycan; NCTP, Na^+^-taurocholate co-transporting polypeptide pol, polymerase; TP, terminal protein.

The circular partially double stranded HBV genome encodes 4 genes— polymerase (P), surface (S; pre-S1 and pre-S2), precore/core (C) and the X protein, encoded by discrete open reading frames (ORFs; Figure 2*A*).^35^, ^36^, ^37^, ^38^ HBV produces 5 viral RNA transcripts of varying lengths, which are translated into 7 distinct proteins (Figure 2*A* and *B*).

This basic genomic organization is common to all hepadnaviruses, although the X gene is absent from most *Avihepadnavirus* species (with the exception of a vestigial X gene in duck hepadnaviruses).^39^ The compact nature of hepadnavirus genomes, which have multiple overlapping reading frames, results in approximately two thirds of nucleotides encoding more than 1 functional element.^40^ This genome structure encompasses virus genes and regulatory regions and restricts redundancy within coding regions (Figure 2*A*). One specific example is the N-terminal region of the precore protein, which is highly conserved among *Orthohepadnavirus* species, likely owing to constraints from the overlapping encapsidating signal (epsilon) sequence.^41^ The negative-sense genomic DNA strand (complementary to the mRNA transcript) is the complete strand—it is held in a circular conformation by an overlap at the 5′ end of the genome (ranging from 50 bp in *Avihepadnavirus* species to 240 bp in *Orthohepadnavirus* species).

Partially double stranded relaxed circular (RC-DNA) in HBV virions is converted into covalently closed circular DNA (cccDNA) inside the hepatocyte nucleus by the viral polymerase filling in the partially single-stranded region of the genome (Figure 2*B*). Biogenesis of cccDNA, including the exact mechanism of DNA repair of the partially single-stranded DNA region of the RC-DNA, is not fully understood. It is likely that cell enzymes such as tyrosyl-DNA phosphodiesterase 2 contribute to cccDNA formation through cleavage of the HBV P from RC-DNA.^42^, ^43^ The viral cccDNA is extremely stable and persists in the nucleus as a viral minichromosome^44^ for the lifespan of the cell, providing the transcriptional template for all RNA species that are translated into viral proteins (Figure 2*B*).

In addition to persisting as a minichromosome in the form of cccDNA, hepadnavirus DNA also integrates into the host genome.^45^ In woodchucks, integration usually occurs within Myc proto-oncogenes, eventually causing HCC in almost all infected animals.^45^ In humans, HBV integration can occur in different sites within the genome, and the consequences are less clear, although chronic HBV infection is associated with liver cancer.^46^ After integration of HBV DNA into the host genome, only the S gene typically remains under the control of its native promoter,^45^ leaving these integrated genomes as a source of HBV surface antigen (HBsAg) production.^47^

During infection, infectious viral particles containing HBV genomes are secreted from infected hepatocytes, in addition to smaller subviral particles and long tubular filamentous particles. These particles are empty shells formed from the HBsAg—they lack a capsid and virus genome and are therefore noninfectious.^48^ The particles typically outnumber infectious virions by as much as 100,000-fold^48^ and are believed to be involved in immune evasion by binding neutralizing antibodies^49^ and potentially promoting T-cell anergy.^50^ Similar particles have been documented in the woodchuck HBV model,^51^ indicating a common role in *Orthohepadnavirus* infections.

## Virus Genotypes and Reference Sequences

Nine different HBV genotypes (A–I) have been defined by >8% divergence at the nucleotide level; a 10th putative genotype (J) was characterized after isolation from 1 individual.^52^, ^53^ The HBV genotypes are further divided into at least 35 subtypes by >4% divergence, with wide variation observed in the numbers of subtypes described per genotype (Table 1).^20^, ^54^, ^55^, ^56^

**Table 1:** HBV Subtypes and Genotype Features

Genotype	Subtypes	Geographic distribution	Genome length (bp)	Distinguishing features of HBV sequence^a^
A	A1–A4	Africa, Europe	3221	6-bp insertion in core gene; G1896A^b^ mutations rare; BCP mutations^c^ common
B	B1–B5	Japan, China, Southeast Asia	3215	B1 and B5 are pure strains, whereas B2, B3, and B4 are recombinants with genotype C in the core region
C	C1–C16	Southeast Asia, Australia	3215	BCP mutations^b^ common
D	D1–D7	Worldwide, Middle East, West Africa	3182	33-bp deletion in pre-S1
E	No subtypes described	West Africa	3212	3-bp deletion in pre-S1
F	F1–F4	North and South America	3215	G1896A^b^ mutations rare
G	No subtypes described	Central America and Europe	3248	36-bp insertion in core and 3-bp deletion in pre-S1; insertion results in a high level of core expression; stop codons at positions 2 and 28 (G1896A^b^) of the precore protein render it unable to express HBeAg; often found in coinfection with other genotypes that express HBeAg
H	No subtypes described	Central America	3215	G1896A^b^ mutations rare
I	I1–I2 (putative^d^)	Southeast Asia	3215	Evolved as a recombinant of genotypes A, C, and G^56^
J (putative)	No subtypes described	Japan	3182	Single isolate identified in elderly Japanese patient with HCC; highly divergent from other human HBV strains; likely a genotype C–gibbon *Orthohepadnavirus* recombinant^53^

NOTE. Further details about genotypes and subtypes can be found in Rajoriya et al^20^ and Tong and Revill.^55^

BCP, basal core promoter; HBeAg, hepatitis B e antigen.

a Insertions and deletions relative to 3215-bp genome length.

b G1896A mutation introduces a premature stop codon in the precore, resulting in loss of HBeAg expression.

c Basal core promoter mutations at A1762T and G1764A result in decreased HBeAg expression.

d Few sequences of genotype I have been characterized, although the genetic distance between isolates suggests there might be 2 subtypes.^56^

There are substantial differences among genotypes in geographic distribution, transmission mode, and clinical outcomes, including emergence of drug resistance and response to therapy^20^, ^57^ (Table 1). However, the data are incomplete—particularly from low- and middle-income countries.^6^, ^58^ Furthermore, it is difficult to associate differences in disease progression and outcome with HBV genetic sequences vs population behavior, coinfections, exposures to drugs and hepatotoxins, and human genetic factors.^5^ Most studies have focused on small numbers of individuals in relatively restricted areas.^57^ Prospective high-resolution genome-wide association studies of large numbers of patients are required to determine how interactions between human and virus genomes affect outcomes.

Sequence data indicate wide variation in the numbers of subtypes within each genotype, ranging from genotype C, with 16 distinct subtypes,^52^ to genotypes E, G, H, and J, each of which consists of a single subtype (Figure 1*B*).^55^, ^56^ Molecular clock analysis has indicated that genotype C is likely to be the oldest genotype^59^—the large number of subtypes is in keeping with its protracted endemic association with human populations.^60^, ^61^ However, it has been a challenge to study the evolution of HBV, because the lack of temporal structure has confounded molecular clock analyses.^62^ Genotypes F and H, which have a smaller number of subtypes but are highly divergent from other genotypes, might have higher rates of substitution.^63^ Genotype F has higher inter-subtype diversity than other genotypes,^64^ which could be due to the geographic range of the populations it infects—from native Alaskan to Latin American populations.^65^, ^66^, ^67^

Genotypes B and C have been associated with higher rates of vertical transmission than other genotypes.^68^ Genotypes A1,^69^ C,^68^ and F have been associated with earlier progression to HCC (particularly in Alaskan natives).^66^ Routine genotype analysis of HBV in infected individuals in clinical practice has not been recommended by US, Asia-Pacific, or European clinical guidelines,^70^, ^71^, ^72^ largely because results do not affect treatment decisions. However, more recent European and US guidelines recognize that genotype variations are associated with responses to therapy with pegylated interferon alfa. This treatment is not recommended for patients who are negative for the HBV e antigen (HBeAg) and infected with genotype D or E, and different stopping points are proposed for patients infected with genotypes A–D who have not responded to therapy.^15^, ^71^ As new therapies are developed, and genotype and subtype data become more widely available, we will develop a better understanding of the effects of genotype on treatment outcome.

## Reference Genomes

There are few robust molecular biology and comparative bioinformatic studies of diverse HBV strains; most published sequences are from HBV genotypes B and C, which together account for >60% of published full-length genomes (data downloaded August 2017). Universally accepted reference sequence(s) and numbering of amino acid residues provide an important foundation for unifying research efforts. With this in mind, we used previously published HBV protein alignments^73^ as a point of reference for pinpointing the sites of immune epitopes within the HBV genome.^74^

Consistent numbering of the HBV genome is a challenge because of genotype-specific differences in genome length and the circular genome. A unified system would be valuable, similar to that proposed for HCV,^75^ in which numbering is based on a reference strain, and a consistent approach has been proposed for documentation of insertions and deletions. Conventional HBV numbering, based on molecular cloning of the genome, typically uses X02763 (genotype A) or NC_003977.2 (genotype D) as a reference strain and defines the genome origin at an *EcoRI* restriction site (GAA/TCC, with nucleotide 1 starting at T), which is embedded within the overlapping P and S genes.^76^ The presence of this restriction site is hypothetical in many HBV isolates,^77^ and this numbering convention is not always followed. Sequence data must be examined and realigned to ensure consistent numbering.^77^

There are several central databases of HBV sequences, including HBVdb^77^ and HEPseq (http://www.hepseq.org/Public/Web_Front/main.php), and recent studies have reported reference sequences for subtypes of genotypes A^78^ and C.^79^ However, there is no unified set of reference sequences of HBV genotypes and subtypes. This differs from HCV and HIV, for which there are large sequence databases and consistently used reference sequences and supporting resources.^80^, ^81^, ^82^ As the HBV field works progressively toward developing unbiased methods for whole-genome sequencing, the numbers of sequences deposited into such databases is likely to increase considerably. A curated alignment of validated genotype and subtype reference sequences would be a valuable resource for researchers, ensuring that comparative analyses are conducted based on a consistent approach.

## Recombination

Inter-genotype HBV recombination has been reported in situations that range from individual case reports to recombinants that have reached fixation and meet the criteria for classification as separate genotypes (genotype I) or subtypes (B2–B4). Breakpoints for the recombinants are not randomly distributed throughout the genotype. Based on the *EcoRI* numbering convention, breakages tend to occur at sites within nucleotides 1700–2000 and 2100–2300,^83^ possibly because of the decreased between-genotype diversity observed in these regions. In addition to the genotype I recombinant, examples include B and C recombinants in parts of mainland East Asia that are now defined as sub-genotypes B2,^22^^,^^84^ B3,^85^ and B4^85^; C and D recombinants reported from Tibet^83^ and China^84^, ^86^; D and E recombinants reported from different parts of Africa^87^, ^88^; A, C, and G recombinants reported in 2 patients in China—although this is based on sequencing only a 1-kb stretch of the genome^89^; and A and G recombinant sequences identified in a patient with A2 infection^90^ and in several patients in Canada.^91^

Intra-genotype HBV recombinants also have been described; genotypes B, C, and E have an increased frequency of intra-genotype recombinant strains compared with other genotypes.^64^ For genotypes B and C, this likely reflects their long association with humans.^59^ Well-defined subtype reference strains could be useful to identify recombination events, to explain current distribution and diversity of viral variants, and to predict future evolutionary directions of the epidemic.^92^

## Approaches to Deep Sequencing

Deep sequencing analyses can increase our understanding of HBV diversity and evolution, control by the immune response, resistance to treatment, and disparities in clinical outcomes. After the success of second-generation short-read sequencing by synthesis approaches (such as the Roche 454 platform [Hoffmann-La Roche, Basel Switzerland] and Illumina [San Diego, CA]), third-generation long-read sequencing technology is advancing (sequencing approaches summarised in a supplementary table on-line at: https://doi.org/10.6084/m9.figshare.7106288). Oxford Nanopore Technologies (Oxford Science Park, UK) provides a radically different approach by generating sequence data in real time directly from samples. It produces complete genomic haplotypes, albeit with some constraints because of the high rate of errors in base-calling algorithms.

NGS studies have the potential to increase our understanding of viral diversity. For example, these studies have detected minor variant populations at low levels^24^, ^25^ and associated quasispecies diversity with treatment outcome and HBeAg status.^27^ There are several factors that have hampered our understanding of the nature and effects of intra-host diversity of HBV. Few studies have used whole-genome sequencing analyses, and sequence output can be biased by the need for prior DNA amplification (especially when viral loads are low) and by representation of the RC-DNA reservoir rather than cccDNA sequences. Studies of HCV have found that diversity in different regions of the genome can indicate contrasting biological processes.^93^, ^94^

In regions of the world where HBV is endemic and mixed infections are common, it can be a challenge to differentiate between coinfection and true recombination using current sequencing approaches.^92^ Several NGS platforms, including Illumina and Roche 454, rely on short reads and amplicon-based approaches, respectively. Therefore, full-genome reconstruction of individual quasispecies can be difficult; inference when multiple genotypes are detected can be unclear.^28^ The development of new long-read sequencing technologies such as those from Oxford Nanopore Technologies and Pacific Biosciences (Menlo Park, CA) will enable more accurate haplotype reconstruction and increase the specificity with which recombinant strains can be distinguished from mixed infection.^95^

## Virus Evolution and Diversity

**Figure 3:**
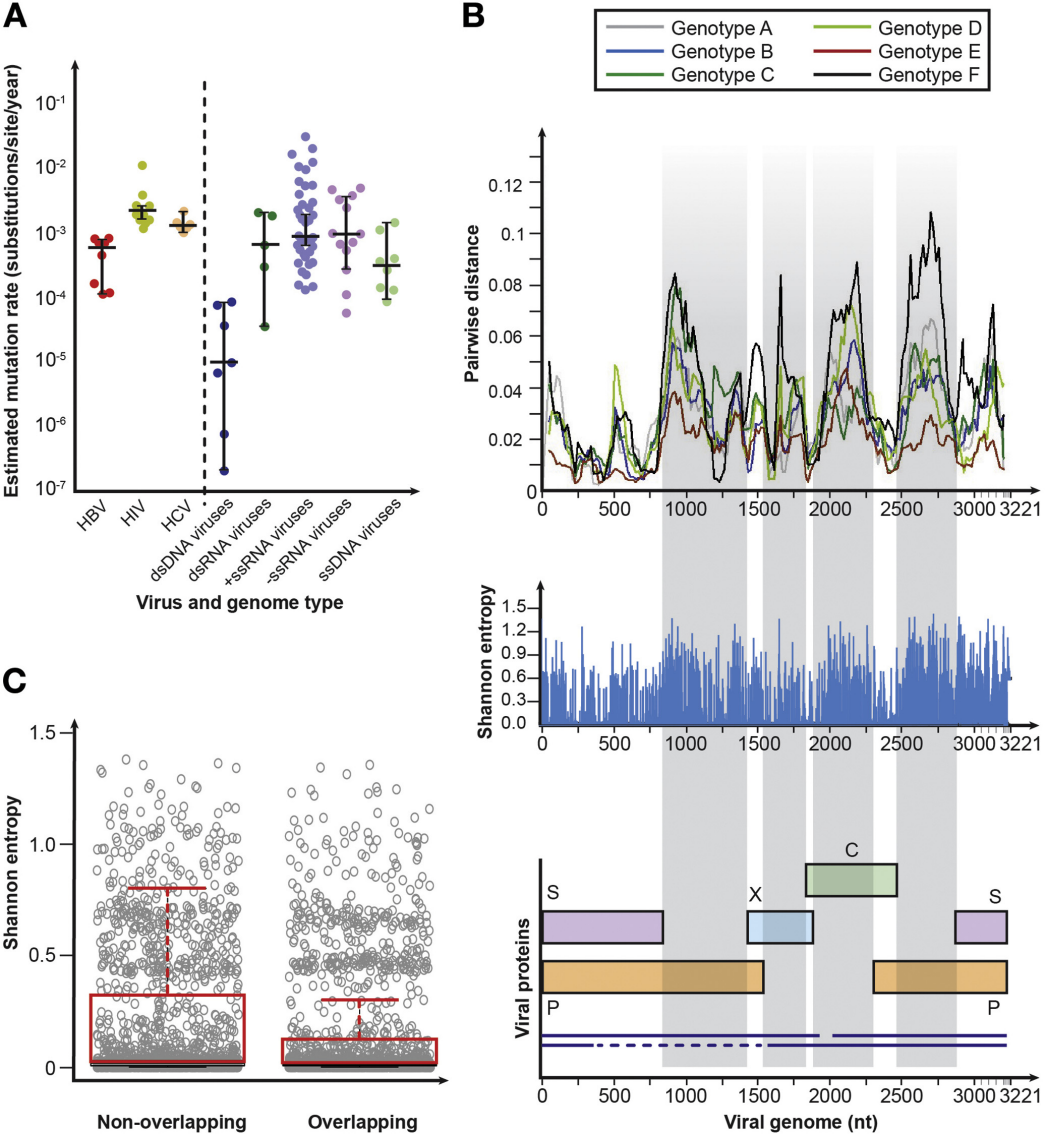
HBV diversity. (*A*) Relation between genome type and substitution rate. Estimates of evolutionary rate (substitutions per nucleotide per year) were taken from Sanjuán^99^ and were calculated using Bayesian molecular clock approaches. For the different genome types, median rates of evolution were 9.32 × 10^−6^ (interquartile range [IQR], 7.00 × 10^−7^–7.20 × 10^−5^) for dsDNA, 6.36 × 10^−4^ (IQR, 1.60 × 10^−4^–1.88 × 10^−3^) for dsRNA, 1.10 × 10^−3^ (IQR, 4.52 × 10^−4^–2.69 × 10^−3^) for +ssRNA, 9.17 × 10^−4^ (IQR, 3.55 × 10^−4^–3.40 × 10^−3^) for −ssRNA, and 2.08 × 10^−4^ (IQR, 1.36 × 10^−4^–5.65 × 10^−4^) for ssDNA. (*B*) Distribution of diversity along the HBV genome. Full-length HBV genome sequences were obtained from HBVdb^77^ in August 2017 (n = 5383). Sequences were aligned using MAFFT (https://mafft.cbrc.jp/alignment/server/).^100^ Sequences for each genotype were randomly shuffled using a function within SSE 1.3^101^ and 250 sequences of each genotype were randomly selected for analysis to normalize the number of sequences of each genotype analyzed. Only 225 sequences were available for genotype F; genotypes G, H, I, and J were excluded from the analysis because there were insufficient numbers of sequences available for comparison with other genotypes. Within-genotype pairwise nucleotide distances were calculated for genotypes A–F using SSE 1.3 using a window size of 150 bp and increments of 20 bp. The greatest variability (typically >5% sequence divergence) is observed in regions where there are no overlapping ORFs. Entropy at each nucleotide within the dataset was calculated using SSE 1.3. (*C*) Comparison of Shannon entropy at each site of overlapping and nonoverlapping regions of the HBV genome. Genotypes were analyzed individually and regions of the genome were divided into overlapping and nonoverlapping regions using an annotated genome (https://hbvdb.ibcp.fr/HBVdb/HBVdbGenome). Mean Shannon entropy in overlapping regions is significantly lower at 0.16 (95% confidence interval, 0.14–0.17) than in nonoverlapping regions (0.20; 95% confidence interval, 0.18–0.21; *P* < .0001 by Mann-Whitney *U*-test). C, core; dsRNA, double-stranded RNA; HCV, hepatitis C virus; ssDNA, single-stranded DNA; ssRNA, single-stranded RNA.

The unique combination of a DNA genome coupled with multiple overlapping ORFs, an RT step, and a stable cccDNA reservoir leads to a complex and unique replicative process. On the one hand, there is evidence that HBV has a relatively low mutation rate (0.0005 substitutions per site per year) compared with other RT viruses—for example, its rate of mutation is 5-fold less than HIV (0.003 substitutions per site per year)^96^, ^97^, ^98^ (Figure 3*A*).^77^, ^99^, ^100^, ^101^ On the other hand, HBV is more diverse than other dsDNA viruses (Figure 3*A*) with a level of variation and rate of evolution that is more comparable to an RNA virus than a DNA virus.^102^ This unusual replication cycle and genomic structure make it difficult to estimate a genome-wide rate of virus evolution.

There is considerable variation in rates of HBV evolution.^62^, ^103^ Faster rates of evolution have been observed in individuals with chronic infection, over specific time periods, or in analyses of families.^103^ Greater diversity has been observed in HBeAg-negative infection.^97^ The long-term rate of HBV evolution is lower than rates reported from short-term studies.^62^, ^63^ For example, sequences of HBV isolated from 2 sets of 400-year-old mummified remains from Korea and Italy^62^, ^104^ had minimal genetic divergence from modern HBV sequences.

Overlapping ORFs can offer a fitness advantage to viruses with high rates of mutation, because substitutions in these regions have higher odds of producing detrimental effects.^105^, ^106^ In addition, there is evidence that many substitutions that occur in the viral genome during the course of chronic infection might not generate variation, but are reversions back to the genotype consensus.^107^ Therefore, most substitutions in the HBV genome are not maintained over the long term.

The rate of substitution in overlapping regions of the HBV genome is 40% lower than in nonoverlapping regions^63^ (Figure 3*B*), and there is a significant difference in entropy between these regions (Figure 3*C*). Overlapping regulatory elements and encoded RNA secondary structures required for replication with the ORFs provide further constraint to nucleotide substitution in the HBV genome. For example, diversity within genotypes in the nonoverlapping region within the X gene is decreased relative to the other nonoverlapping regions (Figure 3*B*), most likely a result of overlap with the basal core promoter region, a regulatory element of the genome that controls expression of precore mRNA and pre-genomic RNA.

Many HBV genotypes have an unexpectedly high level of diversity at the start of the S gene, although this region overlaps with the P gene. Intriguingly, this divergent region of the P–S overlap (often referred to as the spacer region in Pol) has a pattern of codon use that is distinct from the 3′ two thirds of the overlap.^35^ It has been proposed that the P sequence in the 5′ region of the P–S overlap evolved independently,^108^ and that mutations and deletions in this region do not greatly affect the function of the encoded polymerase.^109^, ^110^ This region corresponds to a hydrophilic region under strong immune pressure on the overlapping S gene, indicating that the spacer region of P allows conformational adaptability under selective pressures.^111^ Analysis of HBV sequences from a family transmission network found the precore and middle region of the S gene to be hotspots for sequence diversity compared with the relative conservation of these regions between genotypes.^112^ Opposing effects on HBV genetic diversity are presented in Table 2.^43^, ^63^, ^64^, ^74^, ^102^, ^113^, ^114^, ^115^, ^116^, ^117^, ^118^, ^119^, ^120^, ^121^, ^122^, ^123^, ^124^, ^125^

**Table 2:** Determinants of Diversity and Conservation Within HBV

HBV attribute	Factors associated with sequence conservation	Factors associated with sequence diversity
Genome structure and sequence	Overlapping ORFs and regulatory regions in viral genome (Figures 2*A* and 3*B*) impose constraint on viral plasticity^102^ because nonsynonymous mutations have to be accommodated within 2 different proteins to be viable; this makes most mutations disadvantageous,^113^ an example of constrained evolution.^114^ This is highlighted by greater diversity in nonoverlapping regions (Figure 3*B* and *C*). Examples include the highly conserved epsilon sequence, which overlaps the unique N-terminus of the precore gene and A–T nucleotide substitution at position 1858; it constrains selection of G1896A precore start codon mutation on the opposing strand.^115^	Redundancy within the third codon position in regions where ORFs overlap allows selection of mutations (Figure 3*C*). Host immune responses select escape mutations (eg, within or flanking T-cell epitopes).^74^ Exposure to antiviral therapy selects drug resistance mutations.^116^ Selection of G1896A precore stop codon and BCP mutations.^117^
Persistence and transmission	Superior transmission potential of wild-type variants.^118^ Transmission bottlenecks limit diversity at onset of new infections.^119^, ^120^	Long duration of infection can generate diverse quasispecies populations within hosts.^119^, ^120^, ^121^
Replication cycle	Stable reservoir of cccDNA with long half-life (Figure 2*Bv*); estimated cccDNA half-life 33–57 d in duck hepadnaviruses.^43^ In humans, average cccDNA half-life has been estimated at 9.2 mo but differed markedly in HBeAg-positive (8.6 mo) and HBeAg-negative (26.2 mo) individuals.^122^ Studies are needed to determine half-life in humans at different stages of disease progression.	Error-prone viral RT enzyme with high substitution rate when transcribing pgRNA into RC-DNA (Figure 2*Bvii*). HBV is produced at high rate of replication.^123^
Genotypes	Lowest level of diversity is observed in genotype E (Figures 1*B* and 3*B*), where there is only a single reported subtype.	Increased diversity is a feature of some specific genotypes; genotype F diverges considerably from other genotypes^124^ and shows a high level of inter-subtype diversity^,^^64^, ^125^ (Figures 1*B* and 3*B*). Adaption to a genetically diverse population at some point in the evolutionary history could explain the increased substitution rates.^63^

BCP, basal core promoter; HBeAg, hepatitis B e antigen.

As with determination of genotype, baseline testing for drug resistance is rarely performed. The prevalence of mutations in HBV that cause resistance to treatment varies worldwide, from <2% in the United States and Canada^126^ to >20% in some African cohorts,^116^ but more data are needed. In areas where many individuals also have HIV infection, many patients have been exposed to antiretroviral therapy, including the nucleos(t)ide analogues lamivudine and tenofovir. Prior exposure to antiretroviral therapy could increase the risk for mutations in HBV that mediate resistance to treatment—particularly to lamivudine, which has a low barrier to resistance. Although strains of HBV that are resistant to tenofovir have been described, they are unusual and their effects on patient outcome are not clear.^116^ Therefore, tenofovir is often recommended after nonresponse to alternative therapies.^15^, ^127^

### Within-Patient Diversity

Although some regions of the HBV genome are highly conserved, there are few data on intrapatient diversity. Simultaneous, competing evolutionary pressures can create different subpopulations of HBV within patients (quasispecies) and at the population level. These can produce a more diverse RC-DNA population and a less diverse and stable cccDNA population, with different sequence polymorphisms potentially archived in the cccDNA pool^128^ (Figure 2*Bv*).

To be stably fixed in the virus population, mutant genomes egressed in virions must effectively compete with circulating wild-type viruses to infect hepatocytes and generate cccDNA. This unique population structure maximizes the potential pool of mutants, enabling advantageous virus adaptation within each patient and still eliminating viruses with deleterious mutations. Some less-fit RC-DNA genomes might persist by bypassing egression and being recycled directly back into the nucleus to replenish the viral cccDNA population. However, this model was based on observations from the duck hepadnavirus^129^ and has not been clearly documented in human HBV infection.^130^

Certain HBV polymorphisms and deletions have been associated with specific clinical outcomes, such as cirrhosis and HCC. Examples include diversity in the pre-S region, which has been correlated with progression from chronic HBV infection to HCC.^131^ However, deletions in the pre-S region have been associated with HCC in patients infected with HBV genotypes B and C (in particular, pre-S deletions of nt 2977–3013 in HBV genotype C).^132^, ^133^ Large deletions that result from splicing of the HBV pre-genomic RNA are associated with advanced liver disease, including cirrhosis^134^, ^135^ and HCC.^136^ Mutations in the basal core promoter (A1762T and G1764A), detected by pyrosequencing, have been associated with increased risk of disease progression to cirrhosis or HCC in some populations^137^—mostly in patients with HBV genotype B or C infection—independent of viral load.^138^, ^139^ Likewise, viral diversity is likely to affect response to treatment, although this relation is not clearly defined. A large study correlated a higher level of virus diversity (particularly in the basal core promoter and precore regions) with a lower probability of HBsAg loss.^117^ Other studies have associated HBV heterogeneity with positive effects of treatment.^140^, ^141^

A wide range of measures can be used to assess the diversity in HBV in infected individuals. Broad estimates of virus diversity have been made using pairwise and entropy-based measures,^142^ detection of minor variant viral populations with specific polymorphisms (often associated with drug resistance),^26^, ^28^, ^143^ and detection of mixed-genotype or subtype infections.^28^, ^92^ It is important to increase our understanding of quasispecies dynamics if we are to better understand how selection and fixation of polymorphisms affect patient outcomes, including virus resistance to drugs and vaccines, the antivirus immune response, and development of chronic liver disease. Diverse virus populations could arise through immune selective pressure; there is a balance between the benefit of immune-escape mutations and the deleterious effects of mutations on HBV fitness or replicative capacity.

A study of mother–child pairs demonstrated a relatively tight bottleneck at transmission, with limited virus diversity in infected children compared with their mothers—suggesting only a proportion of HBV strains in the mother are transferred to the child.^119^, ^144^ In mothers with HBV and HIV coinfection, minor HBV variants may be established as the dominant virus in their infants. Mutations in HBsAg were frequently observed in these strains,^145^ indicating that HIV infection opens the HBV transmission bottleneck. Analyses of intrahepatic quasispecies demonstrated an association between intrahepatic diversity (focused within T-cell epitopes) and off-treatment control, indicating a role for immune-mediated selection pressure in control of viremia.^119^ Similarly, increased diversity of quasispecies has been associated with effective therapy,^141^, ^146^ although this observation has not been consistent.^117^

For other blood-borne virus infections, intrapatient virus diversity has been associated with strong suppression by treatment^93^, ^147^ or conversely with poor patient outcome.^94^ However, studies of factors that affect the diversity of HBV are confounded by factors such as genotype and subtype, small heterologous cohorts, variations in sequencing methods, and examination of different areas of the genome. Therefore, it is a challenge to uncover true associations. Studies also are confounded by the geographic distribution of genotypes and the ethnicity of affected individuals.

## Future Directions

Chronic HBV infection is a fundamental global public health challenge for the 21st century. There is not enough unbiased generation and interpretation of sequence data or attempts to unify such data with relevant resources (such as genome annotation, reference sequences, and robust linked clinical data). The development of unbiased and meta-genomic pipelines, alongside carefully collated host metadata, has begun to affect management of patients with infectious diseases.^148^, ^149^, ^150^ Although deep sequencing approaches have not been robustly applied to HBV, there are several situations in which NGS data could be of substantial value, such as in development of diagnostic tools, selection of treatment, analyses of transmission, and studies of HBV pathogenesis.

In virus diagnostics, NGS could be used to identify known or novel viruses or to exclude infectious etiology of clinical syndromes.^151^, ^152^ Previously unrecognized HBV coinfection was detected using a meta-genomic approach in a cohort of patients with acute liver failure,^151^ and new splice variants were identified using Pacific Biosciences technology.^153^ At the same time, NGS might be used to identify existing and new drug-resistant mutations and study their dynamics.^117^, ^154^, ^155^, ^156^, ^157^

Strategies are in development to bring genome sequence analysis to the clinical virology laboratory.^158^ For example, pre-S deletion patterns, combined with quantitative NGS data and machine learning methods, might be used to identify patients at risk for liver disease progression.^133^, ^137^ NGS also might be used to characterize the vertical transmission bottleneck and identify and track outbreaks in a range of settings.^159^, ^160^, ^161^

Challenges remain in the widespread application of NGS platforms, including the need to deplete host reads, which could require enrichment and amplification steps (particularly in detecting viruses at low copy numbers). Systems and reagents are expensive, and interpretation of NGS data requires considerable bioinformatic support and adaptation for different genomic configurations. For HBV, this means refining methods for a circular and partial dsDNA genome. However, the development of portable, real-time, third-generation sequencing platforms, such as the Nanopore MinION (Oxford Nanopore Technologies),^158^ have made the prospect of deep sequencing as a point of care test increasingly feasible. Relatively short and simple sample preparation protocols, minimal setup requirements (a laptop computer), and low costs relative to convention benchtop sequencers make the technology particularly appealing for resource-limited settings. Although the error rate of Nanopore has been too high for robust application to studies of pathogen diversity, rapid improvements are being made to laboratory and bioinformatic protocols.^162^

Substantial gaps remain in our understanding of the relationship between HBV genome structure, replication cycle, diversity, transmission, and clinical outcomes. Recent sequencing advances offer an enormous opportunity to generate datasets that can help to address some of these questions. The generation of standardized reference genomes of all HBV genotypes and subtypes to enable robust and consistent collation and analysis is required to develop insights into current and future epidemiology, to inform better clinical assessment and prognostication, to improve deployment of current antiviral drugs and vaccines, and to drive discovery of new therapeutic agents.
